# Artificial neural network analysis of the oxygen saturation signal enables accurate diagnostics of sleep apnea

**DOI:** 10.1038/s41598-019-49330-7

**Published:** 2019-09-13

**Authors:** Sami Nikkonen, Isaac O. Afara, Timo Leppänen, Juha Töyräs

**Affiliations:** 1 Department of Applied Physics, University of Eastern Finland, Kuopio, Finland; 2 Department of Clinical Neurophysiology, Diagnostic Imaging Center, Kuopio University Hospital, Kuopio, Finland; 3 School of Information Technology and Electrical Engineering, The University of Queensland, Brisbane, Australia

**Keywords:** Neurophysiology, Medical research

## Abstract

The severity of obstructive sleep apnea (OSA) is classified using apnea-hypopnea index (AHI). Accurate determination of AHI currently requires manual analysis and complicated registration setup making it expensive and labor intensive. Partially for these reasons, OSA is a heavily underdiagnosed disease as only 7% of women and 18% of men suffering from OSA have diagnosis. To resolve these issues, we introduce an artificial neural network (ANN) that estimates AHI and oxygen desaturation index (ODI) using only the blood oxygen saturation signal (SpO2), recorded during ambulatory polygraphy, as an input. Therefore, hypopneas associated only with an arousal were not considered in this study. SpO2 signals from 1692 patients were used for training and 99 for validation. Two test sets were used consisting of 198 and 1959 patients. In the primary test set, the median absolute errors of ANN estimated AHI and ODI were 0.78 events/hour and 0.68 events/hour respectively. Based on the ANN estimated AHI and ODI, 90.9% and 94.4% of the test patients were classified into the correct OSA severity category. In conclusion, AHI and ODI can be reliably determined using neural network analysis of SpO2 signal. The developed method may enable a more affordable screening of OSA.

## Introduction

Obstructive sleep apnea (OSA) is a breathing disorder in which the upper airways collapse repetitively during sleep, causing breathing cessation events^1^. The event is called an apnea if the airways are completely obstructed and a hypopnea if the obstruction is partial^1^. OSA is associated with several severe health consequences such as stroke and heart failure^2^. In addition, OSA causes sleep fragmentation and degrades the quality of sleep, which often leads to excessive daytime sleepiness, cognitive impairment, depression, and an increased risk of traffic accidents^3–5^. The prevalence of OSA has been estimated to be as high as 23.4% in females and 49.7% in males^6^.

The diagnostics of OSA is mainly based on the apnea-hypopnea-index (AHI), which is simply the number of apneas and hypopneas per hour of sleep^7^. Alternatively, the oxygen desaturation index (ODI), *i*.*e*. the number of oxygen desaturation events per hour, is sometimes used in OSA screening instead of AHI^8,9^. The severity of OSA is classified into one of four categories based on AHI: No OSA (AHI < 5), mild OSA (5 ≤ AHI < 15), moderate OSA (15 ≤ AHI < 30) and severe OSA (AHI ≥ 30)^7^. Currently, accurate diagnostics of OSA requires manual scoring of apneas and hypopneas from ambulatory polygraphic or in-lab polysomnographic (PSG) recordings, which is time consuming and labor intesive making it an expensive process^10,11^. Most analysis software offer the possibility of automatic scoring of respiratory events, but the accuracy of these automatic scoring algorithms has been shown to be relatively poor. For example, the mean difference between manually and automatically scored AHIs (scored with the ApneaLink-system) was reported to be 5.8 events/hour, indicating underestimation of AHI by the automatic software^12^. Similar findings (the difference of 8.4 events/hour) have been reported with the Embletta-system^13^. In addition, current recording equipment requires multiple sensors that record several signals (e.g. breathing and electroencephalography) further increasing the cost and complexity of OSA diagnostics. Furthermore, due to the cost and the amount of manual work required for accurate scoring, OSA diagnosis is only based on a single recording night. However, several studies have shown that inter-night variations of AHI and ODI are relatively large and therefore even with perfect scoring, a single night’s recording may not be sufficient for accurate diagnosis^14–17^.

Furthermore, the unnatural environment of the sleep laboratory, or even ambulatory diagnostic equipment, can cause discomfort to patients, reducing sleep efficiency and altering the diagnosis, especially on the first night of study^14,18^. Therefore, even with expensive in-lab PSG and careful manual scoring, many patients are misdiagnosed. In fact, it has been estimated that only 7% of women and 18% of men suffering from OSA have diagnosis^19^. There is also a considerable variation between scorers and thus the diagnosis is dependent on the person scoring the recording^20^. In some cases, the diagnosis for the same individual has varied from no OSA to severe OSA^20^. For these reasons, an accurate and automatic estimation of OSA severity could considerably improve its diagnostics.

Blood oxygen saturation signals have been previously used to classify the severity of sleep apnea using various different methods including neural networks^21–26^. However, the number of test subjects in these previous studies has been relatively small^23–25^. Additionally, the methods presented in these studies have not estimated AHI, the accuracy of the classifier has been only modest or a non-standard OSA severity classification has been used, *i*.*e*. only a binary classification with arbitrary thresholds^22–26^. Therefore, in the present study, we aim to introduce an artificial neural network method that would directly estimates AHI and ODI exclusively from the blood oxygen saturation signal.

To the best of our knowledge, the present study is the first artificial neural network- based approach for automatic and accurate estimation of AHI and ODI using only a blood oxygen saturation signal. By first estimating the numeric values of AHI and ODI and determining the severity category of OSA based on those values, a more accurate estimation of the OSA severity can be achieved beyond the severity classification. In addition, the determination of the numeric value of AHI enables the neural network-estimated OSA severity to be directly compared to OSA severity assessed by standard manual scoring.

## Results

We trained two neural networks that utilize oxygen saturation signal as the input, one for the estimation of AHI and one for the estimation of ODI. The differences between manual and neural network estimated AHI and ODI were small in the primary test set. The median absolute error was 0.78 events/hour for AHI and 0.68 events/hour for ODI. All calculated errors between the AHI and ODI estimated by the neural networks and the AHI and ODI determined with the home sleep apnea test (HSAT) in the primary test set are presented in Table 1. The mean square error performance (events/10-minute epoch) of the training set was 1.53 for the AHI-network and 1.27 for the ODI-network while the performance of the validation set was 1.60 for the AHI-network and 0.93 for the ODI-network.

**Table 1:** The differences between values of AHI and ODI determined with manual scoring of polygraphic recordings and automatic artificial neural network analyses in the primary test set and in the Embletta test set.

Error parameter	Primary test set (N = 198)		Embletta test set (N = 1959)	
	AHI	ODI	AHI	ODI
mean absolute error (events/hour)*	1.41	1.17	2.23	1.34
median absolute error (events/hour)*	0.78	0.68	1.35	0.76
min error (events/hour)*	0	0	0	0
max error (events/hour)*	9.45	8.36	40.1	34.5
median % error*	15.0	14.5	25.6	10.1
misclassified: mean absolute error (events/hour)	1.72	1.18	3.40	1.86
misclassified: median absolute error (events/hour)	1.10	0.67	1.83	0.95
misclassified: median % error	12.2	11.0	12.1	8.10

Mean absolute error, median absolute error, and median % error were also calculated separately for those patients who were misclassified to a wrong OSA severity category when using the neural network- estimated AHI and ODI. *Denotes that the error was calculated for the whole test set.

**Figure 1:**
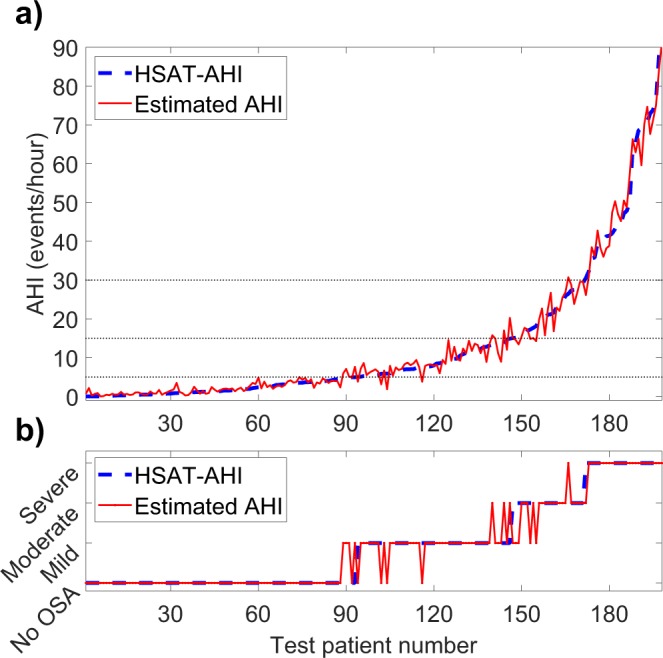
(**a**) Apnea-hypopnea index (AHI) determined with home sleep apnea test (HSAT) and the AHI estimated by the artificial neural network for each test patient in the primary test set (N = 198). (**b**) Obstructive sleep apnea severity classification based on HSAT-AHI values and the severity classification based on neural network estimated AHI.

**Figure 2:**
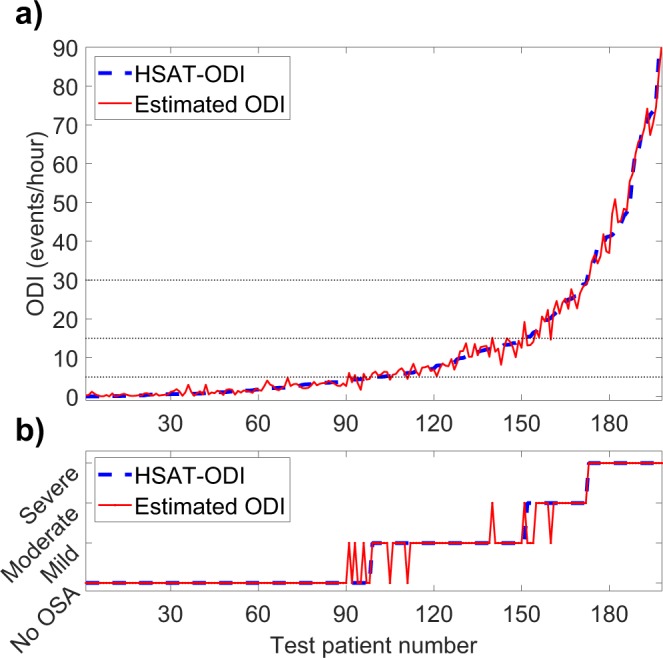
(**a**) Oxygen desaturation index (ODI) determined with home sleep apnea test (HSAT) and the ODI estimated by the artificial neural network for each test patient in the primary test set (N = 198). (**b**) Obstructive sleep apnea severity classification based on HSAT-ODI values and the severity classification based on neural network estimated ODI.

**Figure 3:**
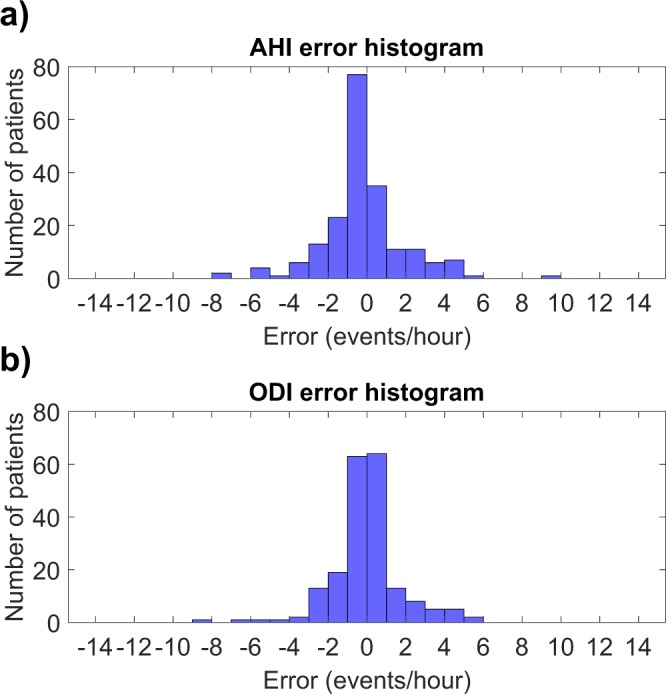
(**a**) Histogram of the absolute errors in AHI estimated by the neural network in the primary test set (N = 198). (**b**) Histogram of the absolute errors in ODI estimated by the neural network in the primary test set (N = 198).

In the primary test set, the AHI estimated by the neural network was close to the HSAT-AHI for all patients although the difference increased slightly with increasing AHI (Fig. 1a). The same was true for ODI albeit with smaller difference between estimated ODI and HSAT-ODI (Fig. 2a). The error distributions for the primary test set are presented as histograms in Fig. 3. Intraclass correlation coefficients were 0.960 (95% CI: 0.947–0.970) between HSAT-AHI and estimated AHI, and 0.975 (95% CI: 0.967–981) between HSAT-ODI and estimated ODI.

**Figure 4:**
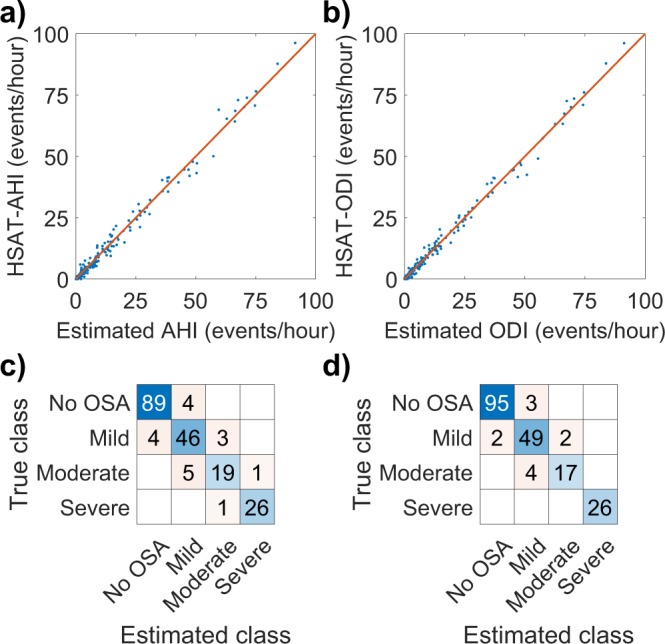
(**a**) Apnea-hypopnea index (AHI) determined with home sleep apnea test (HSAT) vs. the AHI estimated by the artificial neural network in the primary test set (N = 198). The line represents ideal estimation where HSAT-AHI = estimated AHI. (**b**) Oxygen desaturation index (ODI) determined with home sleep apnea test (HSAT) vs. the ODI estimated by the artificial neural in the primary test set (N = 198). (**c**) Confusion matrix for the AHI-network in the primary test set. (**d**) Confusion matrix for the ODI-network in the primary test set.

In the primary test set, 90.9% and 94.4% of the patients were classified to the correct OSA severity category based on neural network -estimated AHI and ODI respectively. This amounts to 18 of the 198 primary test patients being misclassified when using AHI and to 11 being misclassified with ODI (Figs 1b and 2b). Confusion matrixes showing the patient classification by both networks are presented in Fig. 4c,d. HSAT-AHI vs. estimated AHI is presented in Fig. 4a and HSAT-ODI vs. estimated ODI is presented in Fig. 4b.

**Figure 5:**
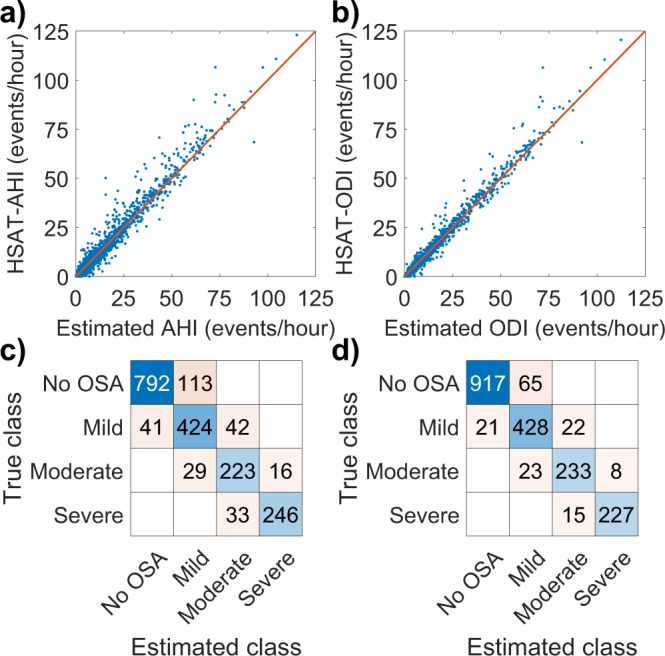
(**a**) Apnea-hypopnea index (AHI) determined with home sleep apnea test (HSAT) vs. the AHI estimated by the artificial neural network in the Embletta test set (N = 1959). The line represents ideal estimation where HSAT-AHI = estimated AHI. (**b**) Oxygen desaturation index (ODI) determined with home sleep apnea test (HSAT) vs. the ODI estimated by the artificial neural in the Embletta test set (N = 1959). (**c**) Confusion matrix for the AHI-network in the Embletta test set. (**d**) Confusion matrix for the ODI-network in the Embletta test set.

The neural network performed well also in the Embletta test set. The median absolute error was 1.35 events/hour for AHI and 0.76 events/hour for ODI. The errors between the HSAT-AHI and HSAT-ODI, and the AHI and ODI estimated by the neural networks in the Embletta test set are also presented in Table 1. 86.0% of patients were correctly classified using AHI and 92.1% using ODI. Intraclass correlation coefficients in the Embletta test set were 0.939 (95% CI: 0.933–0.944) between HSAT-AHI and estimated AHI, and 0.964 (95% CI: 0.961–967) between HSAT-ODI and estimated ODI. HSAT-AHI vs. estimated AHI and HSAT-ODI vs. estimated ODI for the Embletta test set are presented in Fig. 5a,b. Confusion matrixes showing the patient classification by both networks in the Embletta test set are presented in Fig. 5c,d.

## Discussion

The accuracy of our neural networks was high as 90.9% and 94.4% of the patients in the primary test set were classified to the correct OSA severity category based on AHI and ODI respectively. The median absolute errors were also low being just 0.78 events/hour for AHI and 0.68 events/hour for ODI. Only 18 patients were misclassified when using AHI and 11 when using ODI. Most of these misclassified patients had AHI and ODI close to the threshold values between the severity categories where even a small change in AHI or ODI can alter the diagnosis (Figs 1 and 2). However, this problem is encountered in all threshold-based diagnostics and is also an issue when using standard manual scoring. Therefore, the misclassified patients do not necessarily have a major error in their estimated AHI or ODI values. The maximum errors for AHI and ODI in the primary test set were 9.45 events/hour and 8.36 events/hour respectively. These errors are still relatively small considering that the patients with the greatest errors had high AHI values (the patient with the greatest error had an AHI of 69.0 events/hour). As the diagnostic threshold of severe OSA is 30 events/hour, this error would not alter the diagnosis. In addition, none of the test patients had their severity classification differ by more than one severity category i.e., no one changed from mild to severe or from moderate to no OSA for example, as is evident from the confusion matrixes (Fig. 4c,d). This suggests that the neural network was capable of estimating OSA severity at least reasonably well in all of the tested patients, which is also evident from Figs 1, 2 and 4a,b.

The neural networks performed very well also in the Embletta test set as 86.0% of patients were correctly classified using the neural network -estimated AHI and 92.1% using the neural network -estimated ODI. Although the network performed slightly worse in the Embletta dataset, the results are still impressive considering that the dataset was scored by different people and recorded with a different device. These results show that the network generalizes at least reasonably well and can also handle a totally new and unseen dataset.

The values of AHI and ODI can vary significantly (by as much as 1600%) between scorers, especially if they are from different hospitals or sleep laboratories^20^. Considering this large uncertainty in manual scoring, the accuracies of the neural networks developed in this study can be considered excellent. It is also important to note that the HSAT-AHI and HSAT-ODI in this study were determined manually by individual scorers and that other scorers would have come up with different values for the HSAT-AHI and HSAT-ODI.

It is well known that the inter-night variation of AHI and ODI can be large and thus, even with perfect scoring, the severity of OSA cannot be accurately determined from only a single recording night^14–17^. However, due to the high costs of registration and scoring, one recording night is the currently used standard in clinical practice. By using the neural network solution presented in this paper, the diagnostic accuracy could be improved as the patient could be monitored for as many nights as is needed without any extra scoring workload. In addition, since the present neural network only requires a blood oxygen saturation signal, the patients’ sleep efficiency could be improved as it is likely that the oximeter alone does not cause as much discomfort to the patient as the standard polygraphy equipment. It would also allow screening of larger number of patients for OSA since the cost and labor required for diagnosis would decrease significantly.

The neural network models developed in the present study are computationally light and do not require significant resources. For example, analyzing both of the test sets using the neural network models running on a basic personal computer takes less than five seconds, while manual scoring could easily take weeks. The near instantaneous nature of the neural network approach could enable real-time applications for monitoring or as a preliminary estimate for OSA severity before manual scoring. A possible future direction of this study could involve further clinical validation of the presented neural network. The neural network could be used alongside standard manual scoring and the results could be compared to validate the performance of the network also in clinical practice. Furthermore, the neural network could be validated to accurately estimate AHI in different sleeping positions.

## Limitations

The main limitation of this study is that the polygraphic recordings were conducted with ambulatory devices not including the recording of EEG. Therefore, hypopneas associated with an arousal are not included in either the HSAT-AHI or the neural network estimated AHI. In addition, the ambulatory recordings used to train the neural networks are less accurate than full in-lab PSG-recordings. The consequence is that the present neural networks are likely to underestimate the severity of OSA when compared to a full PSG-study. This is especially true for patients who experience only mild desaturations. Therefore, the presented neural network method cannot be used to replace full in-lab PSG studies. It should only be used in cases where ambulatory recording without EEG is enough, such as screening for OSA, where HSAT devices lacking EEG are widely used and accepted^27,28^.

In addition, the data was re-scored according to the 2007 AASM scoring rules, which subsequently have changed slightly. According to the 2007 rules, a desaturation-linked hypopnea is scored if the airflow signal drops ≥30% from the reference level causing at least 4% desaturation while in the current rules (AASM 2012) only a 3% desaturation is required^10,11^. The consequence is that the neural network models were optimized according to the 2007 AASM rules; thus if compared with manual scoring done by the current rules, the networks are likely to slightly underestimate AHI and ODI. This is not a critical issue however, as it could be addressed by re-scoring the whole training set based on the new rules and then re-training the networks. However, with the present dataset, this would require enormous amount of manual labor. Nevertheless, this does not alter the underlying concept of the neural network approach proposed in this study and no significant difference in results is expected if 3% hypopneas were to be included. Another limitation is that the neural network is not able to differentiate between OSA and central sleep apnea (CSA) as this would require information about the breathing effort, which is not present in the SpO2-signal. Additionally, it is possible that the neural network could give a false positive result for a patient who has desaturations not related to sleep apnea. Finally, the neural network is not able to discriminate between patients having REM-dominant sleep apnea and patients having NREM-dominant sleep apnea. In order to achieve this kind of discrimination, the neural network would need to be retrained to estimate AHI during REM and NREM sleep using full PSG recordings allowing an accurate determination of sleep stages. As this cannot be done using the present dataset, further studies are warranted to increase the clinical usefulness of the present neural network.

## Conclusions

In conclusion, it is possible to use neural networks to automatically and accurately estimate AHI and ODI using only the oxygen saturation signal. This automatic approach could allow more patients to be screened for a fraction of the current cost and thus enable treatment for many who are suffering from OSA but are not diagnosed.

## Methods

**Figure 6:**
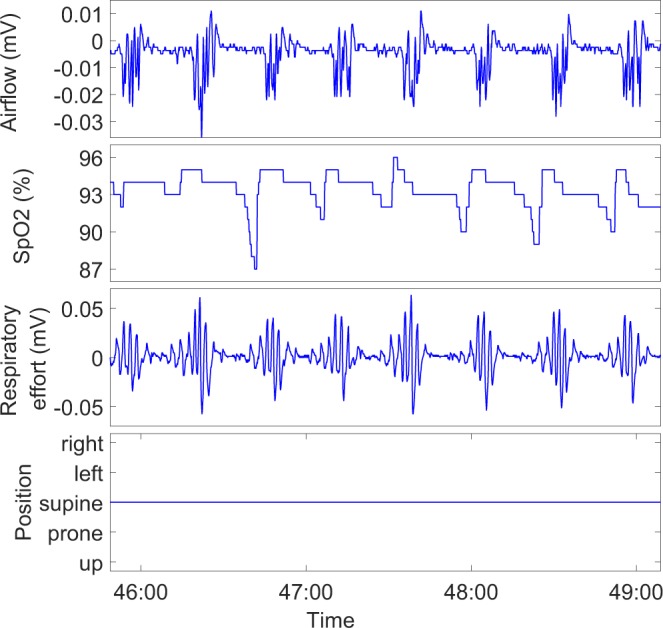
Example of a four channel Unisalkku recording used in the study.

A dataset consisting of 1989 polygraphic recordings of patients with suspected OSA was used to train and validate the neural networks. The data was collected using an ambulatory Unisalkku-device (Neurotech, Kortejoki, Finland) recording four channels (airflow, respiratory effort, body position, blood oxygen saturation) between 1992 and 2003 in Kuopio University Hospital^29^. An example of the recorded signals is presented in Fig. 6. All recordings were manually reanalyzed during 2012–2015 using the American Academy of Sleep Medicine (AASM 2007) scoring rules^10^. As defined by these rules, hypopnea was scored, if the airflow signal dropped ≥30% from reference level causing at least 4% desaturation in the SpO2-signal (AASM 2007 rule 4A)^10^. The research was performed in accordance with relevant guidelines and informed consent was obtained from all participants. Ethics Committee of the Hospital District of Northern Savo, Kuopio, Finland approved the study (127/2004, 24/2013).

The Unisalkku recordings were divided into a training set of 1791 recordings (≈90%) and to a primary test set of 198 recordings (≈10%). The division was done by first sorting all patients based on their AHI and assigning every 20^th^ patient into the test set. This division method resulted in a test set that included patients from all OSA severity categories with close to the full range of AHI. 99 patients from the training set were also randomly selected to a separate validation set leaving a total of 1692 patients for the training set. Patients’ characteristics in the whole Unisalkku dataset, training set, validation set and primary test set are presented in Tables 2, 3 and 4.

**Table 2:** The patient demographic data: median and range for continuous variables in the whole Unisalkku dataset, training set, validation set, primary test set and the Embletta test set.

	Whole Unisalkku dataset (N = 1989)		Training set (N = 1692)		Validation set (N = 99)		Primary test set (N = 198)		Embletta test set (N = 1959)	
	Median	Range	Median	Range	Median	Range	Median	Range	Median	Range
Age (years)	48.1	18.3–81.1	48.2	18.3–80.3	46.4	22.4–70.0	48.3	20.9–81.1	49.6	18.1–87.7
AHI (events/hour)	5.3	0.0–148.7	5.3	0.0–148.7	4.8	0.0–101.6	6.0	0.0–99.1	5.9	0.0–123.0
ODI (events/hour)	4.5	0.0–149.0	4.4	0.0–149.0	4.5	0–99.7	5.0	0–98.8	5.0	0.0–120.5
BMI (kg/m^2^)	28.4	17.5–74.0	28.4	17.5–74.0	28.8	18.8–60.4	28.7	17.6–54.2	28.4	17.5–74.0
Minimum SpO2 (%)	80	1–97	81	1–97	81	1–93	79	1–97	86	1–96
Apnea proportion (%)	15.4	0.0–100.0	15.6	0.0–100.0	14.5	0.0–100.0	15.1	0.0–100.0	34.6	0.0–100.0
Time with <90% SpO2 (%)	0.9	0.0–100.0	0.9	0.0–100.0	0.8	0.0–100.0	1.0	0.0–76.7	1.1	0.0–100.0
Supine time (%)	38.5	0.0–99.3	38.5	0.0–98.1	38.6	0.0.–97.6	38.8	0.0–99.1	37.4	0.0–97.3

AHI = apnea-hypopnea index, ODI = oxygen desaturation index, BMI = body mass index, apnea proportion is the proportion of apnea events out of all obstructive (apneas and hypopneas) events, supine time is the proportion of recording time spent in supine position.

**Table 3:** The patient demographic data: number and proportion of OSA severity and known preexisting medical conditions in the whole Unisalkku dataset, training set, validation set, primary test set and the Embletta test set.

	Whole Unisalkku dataset (N = 1989)		Training set (N = 1692)		Validation set (N = 99)		Primary test set (N = 198)		Embletta test set (N = 1959)	
	Number	Proportion	Number	Proportion	Number	Proportion	Number	Proportion	Number	Proportion
**OSA severity**										
No OSA	967	48.6%	827	48.9%	50	48.5%	90	45.5%	905	46.2%
Mild	505	25.4%	430	25.4%	23	25.8%	52	26.3%	507	24.9%
Moderate	257	12.9%	218	12.9%	12	12.6%	27	13.6%	268	13.7%
Severe	260	13.1%	217	12.8%	14	13.1%	29	14.6%	279	14.2%
Hypertension	951	47.8%	799	47.2%	48	48.5%	104	52.5%	—	—
Diabetes	399	20.1%	330	19.5%	20	20.2%	49	24.7%	—	—
Coronary artery disease	247	12.4%	204	12.1%	13	13.1%	30	15.1%	—	—

A ‘—’ denotes that this data was not collected for the Embletta dataset.

**Table 4:** The number of manually scored apneas, hypopneas and desaturation events in the whole Unisalkku dataset, training set, validation set, primary test set and the Embletta test set.

	Events in the whole Unisalkku dataset	Events in training set	Events in validation set	Events in primary test set	Events in Embletta test set
Apneas	58 176	50 042	2 593	5 541	106 314
Hypopneas	125 367	104 721	6 989	13 657	93 996
Desaturation events	169 775	142 827	8 929	18 019	182 265

To test how the neural networks perform in a completely different dataset, we also formed an additional test set consisting of recordings of 1959 suspected sleep apnea patients conducted in the Diagnostic Imaging Center, Kuopio University Hospital during 2004–2015. The recordings were conducted with an Embletta device (Natus Medical Inc., CA, USA) recording seven channels (nasal pressure airflow, thermistor airflow, blood oxygen saturation, thorax respiratory effort, abdomen respiratory effort, audio, body position) and equipped with a Nonin XPOD 3012 pulse oximeter (Nonin Medical Inc., MN, USA). The recordings were scored with the same AASM 2007 scoring rules^10^ as the Unisalkku dataset. This Embletta dataset was utilized only as a test set and was not used for training the network. Patients’ characteristics in the Embletta dataset are also presented in Tables 2, 3 and 4.

The blood oxygen saturation signals from patients belonging to the training and validation sets were divided into 10-minute epochs with 98% overlap and downsampled from a sampling frequency of 4 Hz to 0.5 Hz. We calculated AHI and ODI for each epoch based on the manually reanalyzed recordings. These 10-minute epochs were pooled into one large training dataset (total of 3 480 024 epochs) which was used to train the neural networks. Two separate networks were trained with different targets: one for AHI and one for ODI. The networks consisted of three feedforward layers of sizes 60, 15 and 5. Sigmoid symmetric transfer function was used before each layer. A scaled conjugate gradient backpropagation algorithm was used as the training function^30^. The output variables of the networks were continuous values representing AHI and ODI for the corresponding input epoch. Mean squared error was used as a performance function for the networks. The networks were trained until the validation set performance started decreasing, i.e. the value of mean squared error started to increase for 100 continuous iterations at which point the training was stopped and the network with the best validation set performance was selected. This approach also helps to avoid overfitting the network to the training set. MATLAB (2017b, MathWorks, Natick, MA) with custom functions and MATLAB’s neural network toolbox was used to train the networks.

Since the neural networks were trained to estimate AHI or ODI for a 10-minute epoch, the oxygen saturation signals of patients belonging to both independent tests set were also split into similar 10-minute epochs, downsampled to 0.5 Hz, and used to estimate AHI and ODI of each epoch. The estimated full-night AHI and ODI for each test patient were calculated as an average of the values obtained from the 10-minute epochs.

The mean absolute error, median absolute error, maximum error and median percentage error of the estimated AHI and ODI for all patients in both test sets were calculated to assess the accuracy of the networks. In addition, the estimated values of AHI and ODI were used to classify the test patients into the standard OSA severity categories (no OSA, mild OSA, moderate OSA, severe OSA) and the number and percentage of correctly classified patients were calculated. Classification accuracy was calculated by dividing the number of correctly classified patients by the total number of patients. To quantify the magnitude of errors that lead to misclassification, we also calculated the mean absolute error, median absolute error, and median percentage error separately for patients that were misclassified into a wrong OSA severity category using the neural networks.

Additionally, the intraclass correlation coefficients (ICC) between the HSAT-AHI and estimated AHI and between the HSAT-ODI and estimated ODI were calculated. ICC can be used to assess the consistency of measurements made by different observers, *i*.*e*., in this case the scorers and the neural network^31,32^.
