# Author Correction: Artificial neural network analysis of the oxygen saturation signal enables accurate diagnostics of sleep apnea

**DOI:** 10.1038/s41598-020-62003-0

**Published:** 2020-03-13

**Authors:** Sami Nikkonen, Isaac O. Afara, Timo Leppänen, Juha Töyräs

**Affiliations:** 1 Department of Applied Physics, University of Eastern Finland, Kuopio, Finland; 2 Department of Clinical Neurophysiology, Diagnostic Imaging Center, Kuopio University Hospital, Kuopio, Finland; 3 School of Information Technology and Electrical Engineering, The University of Queensland, Brisbane, Australia

Correction to: *Scientific Reports* 10.1038/s41598-019-49330-7, published online 13 September 2019

This Article incorrectly states that informed consent was obtained. Consent for research was not sought, because under Finnish law (Law on medical research 2 § (23.4.2004/295)) the need for consent is not required for retrospective chart reviews.

